# The Midwives Bill

**Published:** 1902-06-14

**Authors:** 


### The Hospital

A Journal of

The Medical Sciences and Hospital Administration.

Vol. XXXII.—No. 820. Edited by Sir Henry Burdett, K.C.B., and Solomon C. Smith, M.D., M.R.C.P. [June 14, 1902.

### The Midwives Bill.

The Midwives Bill has at last passed the report stage in the House of Commons, and, although one at least of the "amendments" introduced into it on the occasion will probably be again "amended" in the House of Lords, there seems much reason to believe that this process may be accomplished before the termination of the session, and that the re-amended Bill may be converted into an Act by the Royal Assent. It would be difficult to find, even if the foregoing belief should be justified by the event, a more striking evidence than has been afforded by the Bill of the wastefulness and futility of our Parliamentary system, when regarded as a means of procuring any improvement of legislation which does not admit of being used as a means of party conflict or as an agency for "dishing" political adversaries. In the many years which have passed since the state of midwifery practice in England was shown to be a public scandal, urgently calling for legislative treatment, hundreds of lives must have been lost as a direct result of the indifference of our representatives to any question in which nothing more serious than the avoidable mortality of poor women in childbirth, and the consequent orphanage of their children, was involved. Time after time, and session after session, some progress has been made with a greatly needed measure, but no Government has yet felt the care of such a measure to be a duty, and the chatter of any Irish blatherumskite has been permitted to fill up the hours which might have been devoted to the supply of one of the most urgent wants of the poor. How far the clauses which have been at last accepted by the House of Commons will be effectual in dealing with the evils against which they are directed it would, perhaps, be somewhat rash to conjecture. Mr. Ritchie's declaration that it would be better to pass a "moderate" measure as "a first step in the direction generally desired" is not a highly encouraging one.

The principal amendments on the report of the Committee were moved by Mr. T. P. O'Connor and Mr. Ambrose. That of the former gentleman provided for the removal from the midwives' register of any person who had at any time, either before or after registration, been convicted, either in his Majesty's dominions or elsewhere, of an offence which, if committed in England, would be a felony or misdemeanour, or who had been "guilty of any disgraceful conduct in her practice as a midwife;" and threw upon the Midwives Board the duty of "causing inquiry to be made" into any such case, and of taking action upon it in the event of "proof" being obtained. As regards convictions in the United Kingdom, such proof will be easily obtainable from official sources, and might be acted upon safely and without demur; but the question of "disgraceful conduct" stands upon a totally different footing, as to which the experience of the Medical Council might afford some warning to Parliament. Would it be disgraceful conduct for a midwife to be drunk, either during her official ministrations or at other times? and, if so, what would be the definition of drunkenness? and by whose evidence could the fact be established? No charge would be more easy to bring, none would be more difficult to refute; while the practice of midwives, as a rule, would be among people whose personal feelings against or in favour of the accused would be far stronger than their attachment to truth for its own sake. The proposal to establish an irregular judicial tribunal, for the purpose of dealing with undefined and undefinable offences, seems to us to be totally wrong in principle, and to be likely to lead to grave miscarriages of justice in practice. It would be far better to be content with the removal from the register of convicted persons, and to leave those who were only "disgraceful" to be punished by the natural consequences of disgrace. The amendment of Mr. Ambrose, which totally forbids the midwifery practice for gain of unregistered persons, or the assumption by them of any title implying that they are recognised by law, was accepted after some debate, the time for its coming into operation having first been postponed from the year 1905 to the year 1910. It will give the registered midwife a greater amount of legal protection than is given to the registered medical practitioner; and it is not uninteresting to note that Mr. T. P. O'Connor, in the debate, laid down the proposition that "if the law is not strong enough to prevent quacks from practising, it ought to be strengthened." Stated in this way, the proposition certainly includes medicine and surgery as well as midwifery; and we hope it may be taken to express the opinion of many other members of the House. The financial straits of the Medical Council to which we referred last week, will probably compel that body, before very long, to exert itself in an endeavour to procure some amendments of the Medical Acts; and if the Legislature should prove to be sufficiently educated to include among these amendments a really operative prohibition of unqualified practice, the gain to the profession would not be unimportant, while that to the public would be immense. Among the many demoralising influences of our time, few are more widely influential, or more profoundly hurtful to the community, than the tacit sanction given by the law to the false pretences of quacks.

